# A clinical study to determine the threshold of bronchodilator response for diagnosing asthma in Chinese children

**DOI:** 10.1007/s12519-019-00293-9

**Published:** 2019-08-16

**Authors:** Xiao-Hui Kang, Wan Wang, Ling Cao

**Affiliations:** 1 Children’s Hospital Affiliated to the Capital Institute of Pediatrics, No.2 Ya bao Road, Chaoyang District, Beijing, China; 2 Shanxi Provincial Children’s Hospital, Taiyuan, China

**Keywords:** Asthma diagnosis, Bronchodilator response, Threshold

## Abstract

**Background:**

There is few objective, clinically feasible and inexpensive test for diagnosing childhood asthma. We want to find an ideal way to solve it.

**Methods:**

The control group was 301 non-asthmatic children, and the asthma group was 286 asthmatic children. The asthmatic children were divided into three groups according to the severity of their disease. Pre- and post-bronchodilator spirometer tests were performed, and the main spirometer parameters were compared. The bronchodilator response (BDR) [BDR is used to determine the reversibility of airway obstruction by measuring the changes of forced expiratory volume in the first second (FEV_1_) before and after inhalation of bronchodilators] was then determined, and the optimal threshold of BDR for diagnosing childhood asthma was found.

**Results:**

301 non-asthmatic children and 286 asthmatic children participated in the study, the demographics were similar. FEV_1_ for pre-bronchodilator of asthmatic children was significantly lower than that of non-asthmatic children (*P* ≤ 0.01). BDR of non-asthmatic children was 3.30 ± 3.85%. BDR of asthmatic children was 9.45 ± 9.15%. There was no significant difference in BDR for patients with different severities of asthma within the group. BDR had no statistical correlation with gender, age, height, weight in neither non-asthmatic children nor asthmatic children. On the receiver-operating characteristic curve, a BDR threshold of ≥ 7.5% offered an optimal balance in asthma diagnosis with a sensitivity rate of 50.7% and specificity rate of 87.7%. Meanwhile, with a BDR threshold of ≥ 12%, the sensitivity rate was 28.7% and the specificity rate was 96.3%.

**Conclusion:**

A BDR threshold of ≥ 7.5% has more value in childhood asthma diagnosis as compared to ≥ 12%.

**Electronic supplementary material:**

The online version of this article (10.1007/s12519-019-00293-9) contains supplementary material, which is available to authorized users.

## Introduction

The prevalence of childhood asthma in China is increasing significantly. The national statistics in 1990, 2000 and 2010 showed the cumulative prevalence in children under 14 years of age was 1.09% [1], 1.97% [2], and 3.02% [3], respectively. Early identification of these children will allow earlier treatment and improve outcomes.

Using an objective, clinically feasible and inexpensive test will improve the diagnostic level. Challenge test for diagnosing asthma using methacholine, histamine or cold air is expensive and difficult for the regional clinics that provide the bulk of care for children in China. There are not enough resources to diagnose children who had asthma. Studies by Galant et al. [4], Tse et al. [5] and Dundas et al. [6] showed that using bronchodilator response (BDR) rather than the classic challenge test is an effective way of diagnosing asthma in children. They found that BDR ≥ 8% or ≥ 9% as the positive threshold value had the best combination of sensitivity and specificity. These studies did not include native Chinese children, so we want to find the threshold of BDR for diagnosing asthma in Chinese children.

## Methods

The Ethics Committee of Capital Institute of Pediatrics approved the study and signed informed consent was obtained from each child’s parent or legal guardian.

### Control group

Children aged 4–12 years were selected from kindergartens and primary schools located near the Children’s Hospital Affiliated to the Capital Institute of Pediatrics from January 2012 to December 2015. First, leaflets were released to the children’s parents. If they consented to participate in the study, their children would perform the BDR [BDR is used to determine the reversibility of airway obstruction by measuring the changes of forced expiratory volume in the first second (FEV_1_) before and after inhalation of bronchodilators] test at the appointed time and fill in a detailed questionnaire on that day. The original questionnaire is a section of the American Thoracic Society pulmonary disease questionnaire for children under the age of 13 years (AST-DLD-78-C) [7]. Physicians from our department translated the original questionnaire into Chinese. Later other physicians translated the Chinese questionnaire back into English to check the quality and accuracy of the translation.

The inclusion criteria included: (1) subjects were willing to participate in the study, parents signed informed consent; (2) subjects were 4–12 years old; (3) pulmonary physical examination was normal.

The exclusion criteria included: (1) abnormalities of the thorax; (2) previous history of severe cardiac (significant changes in hemodynamics) or lung disease, or other systemic diseases; (3) recurrent episodes of wheezing diseases (such as asthma, bronchiolitis), tuberculosis, pleurisy, history of chronic lung disease; (4) upper or lower respiratory tract infections within the previous 4 weeks; (5) obesity [body mass index (BMI) > 30] [8]; (6) regular smoking for over 1 month (including first-hand and second-hand smoking); (7) a living environment in which they are exposed to harmful gases or smog from serious pollution; (8) poor cooperation.

We sent leaflets to 541 parents whose children were in kindergartens or primary schools nearby. 450 parents agreed to participate in our study. On the appointed day, 53 children coughed, expectorated or were absented. 11 children had obesity or thoracic deformity. Six children could not cooperate with the test. A total of 380 children completed the test. Detailed questionnaires were given to the children, and 23 of them did not answer it. 56 patients with suspected asthma or other lung diseases were excluded. Therefore, there were 301 cases of children who met the requirements and were included in the study as the control group.

### Asthmatic children

Asthmatic children aged 4–12 years were recruited from the Asthma Prevention and Treatment Center of the Children’s Hospital Affiliated to the Capital Institute of Pediatrics, diagnosed by specialists. Diagnostic criteria were used according to the national guidelines for the diagnosis and prevention of asthma in Chinese children (2008 edition) [9].

If the patients were diagnosed with asthma, and fit the inclusion criteria, they were included in the asthma group.

The inclusion criteria included: (1) subjects were willing to participate in the study, parents signed informed consent form; (2) asthma diagnosed by specialists; (3) have not used controller medications in the previous 6 weeks (including inhaled corticosteroids and leukotriene receptor modulators); (4) no symptoms or mild symptoms, no wheezing upon initial visit.

The exclusion criteria included: (1) thorax was abnormal, such as chicken, funnel chest, costal margin eversion, etc.; (2) previous history of tuberculosis, pleurisy, or chronic lung disease, severe cardiac (significant changes in hemodynamics), or other systemic diseases; (3) upper or lower respiratory tract infections within the previous 4 weeks; (4) regular smoking for over 1 month (both first-hand and second-hand smoke); (5) obesity (BMI > 30) [8]; (6) a living environment in which they are exposed to harmful gases or smog from serious pollution; (7) in the past 6 hours, short acting bronchodilators had been used; (8) in the past 24 hours, long-acting bronchodilators had been used; (9) in the past 3 days, systemic steroid had been used; (10) poor cooperation.

There were 306 patients that fulfilled the inclusion criteria, but five patients took relevant medication recently, eight had upper respiratory infection in the last 4 weeks, four had thorax abnormalities, three could not cooperate with the spirometer tests. Consequently, they were all excluded. 286 patients completed the study. According to the classification of asthma severity of Global Initiative for Asthma 2008 [10], asthmatic children were divided into four groups: intermittent status, mild persistent status, moderate persistent status, and severe persistent asthma. Patients were assessed for 1 month to determine their severity.

In this study, we used the Master Screen IOS (made by JARGER in Germany) for testing. All operations and quality control of spirometer testing were referred to Practical Guide to Pulmonary Function testing [11]*.*

All subjects produced at least three technically acceptable spirometer curves (in fewer than 8 attempts). The best curve was recorded for analysis. the main observation parameters included pre- and post-bronchodilator FEV_1_, and the percentage of predicted value of FEV_1_, forced vital capacity (FVC), peak expiratory flow (PEF), average flow rate of 25–75% out of vital capacity (MMEF) and the BDR [calculated as: (FEV_1_ L post-bronchodilator − FEV_1_ L pre-bronchodilator)/FEV_1_ L pre-bronchodilator × 100%]. After the baseline spirometer test, the subjects inhaled 0.5% salbutamol sulfate (GlaxoSmithKline, United Kingdom) using a PARI compressor nebulizing with a mask. For subjects ≥ 5 years old, 1 mL (5 mg) was inhaled. For subjects < 5 years old, 0.5 mL (2.5 mg) was inhaled. 15 minutes after nebulization, spirometer tests were performed again.

### Statistical analysis

Data were analyzed using the SPSS 20.0 software. Student’s *t* tests were used to compare the characteristics of each group, as well as the changes to spirometer parameters before and after bronchial dilation tests and to compare spirometer between asthmatic children and non-asthmatic children. The characteristics of different severity groups of asthma patients were compared with a variance analysis. Linear correlation analysis was performed to evaluate the relationship between the FEV_1_ improvement rate and gender, age, height, and weight. The data were used to plot a receiver-operating characteristic (ROC) curve (this is a comprehensive index reflecting the continuous variables of sensitivity and specificity). It reveals the relationship between sensitivity and specificity by composition. It draws a curve with sensitivity as ordinate and specificity as abscissa. The larger the area under the curve, the higher the diagnostic accuracy represents the sensitivity and specificity of BDR for diagnosing childhood asthma.

## Results

### Basic characteristics

There were 301 normal children as the control group and 286 asthmatic children as the asthma group, the demographic and clinical data of the two groups were consistent. There were no significant differences between the control group and the asthma group in age (*P* = 0.10), height (*P* = 0.27) or weight (*P* = 0.81) (Table 1). There were 79 children younger than 6 years old in the control group and 87 in asthma group, accounted for 26.2% and 30.4% separately.

**Table 1:** The basic characteristics of the non-asthmatic and asthmatic groups

Variables	Non-asthmatic group (*n* = 301)	Asthmatic group (*n* = 286)
Males, *n* (%)	160 (53.2)	194 (67.8)
Age (y), mean ± SD	7.56 ± 2.41	7.22 ± 2.44
Height (cm), mean ± SD	129.61 ± 15.63	128.19 ± 15.75
Weight (kg), mean ± SD	29.65 ± 11.01	29.89 ± 12.27

*SD* standard deviation

### Non-asthmatic group

There were no significant differences in age, height, weight and main spirometer parameters between male and female subjects (Supplementary Table 1). The main spirometer parameters (FEV_1_, FVC, PEF, MMEF) in non-asthmatic children post-bronchodilator were higher than the baseline (Table 2), but the increase is relatively small. And the BDR in non-asthmatic children was 3.30 ± 3.85%. There was no correlation with gender (*r*_s_ = 0.016, *P* > 0.5), age (*r*_s_ = 0.106, *P* > 0.5), height (*r*_s_ = 0.118, *P* = 0.041), or weight (*r*_s_ = 0.143, *P* = 0.013).

**Table 2:** Pre- and post-BD parameters in non-asthmatic and asthmatic group

Parameters	Non-asthmatic group			Asthmatic group		
	Pre-BD	Post-BD	*P*	Pre-BD	Post-BD	*P*
FEV_1_ (L)	1.76 ± 0.58	1.81 ± 0.59	< 0.001	1.48 ± 0.54	1.60 ± 0.57	< 0.001
FVC (L)	1.91 ± 0.65	1.93 ± 0.66	< 0.001	1.78 ± 0.69	1.84 ± 0.69	< 0.001
PEF (L)	4.11 ± 1.26	4.26 ± 1.26	< 0.001	3.37 ± 1.20	4.61 ± 1.20	< 0.001
MMEF (L)	2.07 ± 0.77	2.42 ± 0.83	< 0.001	1.46 ± 0.62	1.79 ± 0.69	< 0.001

Values are mean ± SD. *BD* bronchodilator, *FEV*_*1*_ forced expiratory volume in the first second, *FVC* forced vital capacity, *PEF* peak expiratory flow, *MMEF* average flow rate of 25–75% out of vital capacity, *SD* standard deviation

### Asthma group

At this group, 53.8% (154/286) had intermittent asthma, 35.3% (101/286) had mild persistent asthma, and 10.8% (31/286) had moderate persistent asthma (Supplementary Table 2). PEF decreased more in the moderate group compared to the intermittent group (*P* < 0.05). There was no significant difference in other characteristics between different severity groups (*P* > 0.05). The main spirometer parameters of asthmatic children were significantly improved post-bronchodilator (Table 2). The BDR of asthmatic children was 9.45 ± 9.15%. There was no significant correlation with gender (*r*_s_ = 0.058, *P* > 0.1), age (*r*_s_ = 0.018, *P* > 0.5), height (*r*_s_ = 0.014, *P* > 0.5) or weight (*r*_s_ = -0.054, *P* > 0.1).

### Comparison of spirometer parameters between non-asthmatic children and asthmatic children

Compared with the non-asthmatic children, the main spirometer parameters of the asthmatic children were significantly lower (Table 3). FEV_1_ is 1.76 in the non-asthmatic children, 1.48 in the asthmatic children, and FVC 1.91, 1.78; PEF 4.11, 3.37; MMEF 2.07, 1.47, respectively. Comparing of the improvement to main spirometer parameters post-bronchodilator between non-asthmatic children and asthmatic children, we found FVC improved the least, and MMEF improved the most among spirometer parameters. The main spirometer parameters of asthmatic children all significantly improved compared with non-asthmatic children (Table 4).

**Table 3:** Comparison of pulmonary function parameters in non-asthmatic and asthmatic group

Parameters	Non-asthmatic group	Asthmatic group	*P*
FEV_1_	1.76 ± 0.58	1.48 ± 0.54	< 0.001*
FVC	1.91 ± 0.65	1.78 ± 0.69	0.016*
PEF	4.11 ± 1.26	3.37 ± 1.20	< 0.001*
MMEF	2.07 ± 0.77	1.47 ± 0.62	< 0.001^†^

Values are mean ± SD. *FEV*_*1*_ forced expiratory volume in the first second, *FVC* forced vital capacity, *PEF* peak expiratory flow, *MMEF* average flow rate of 25–75% out of vital capacity, *SD* standard deviation. ^*^P value of Student’s *t* test. ^†^*P* value of rank sum test

**Table 4:** Comparison of the improvement rate of main pulmonary function parameters in non-asthmatic and asthmatic group

Parameters (%)	Non-asthmatic group	Asthma group	*P*
ΔFEV_1_	3.30 ± 3.85	9.45 ± 9.15	< 0.001
ΔFVC	0.89 ± 3.55	4.53 ± 7.86	< 0.001
ΔPEF	4.06 ± 7.54	8.73 ± 11.53	< 0.001
ΔMMEF	19.30 ± 17.53	25.36 ± 24.00	< 0.001

Values are mean ± SD. *FEV*_*1*_ forced expiratory volume in the first second, *FVC* forced vital capacity, *PEF* peak expiratory flow, *MMEF* average flow rate of 25–75% out of vital capacity, *SD* standard deviation, *Δ* improvement rate

**Fig. 1:**
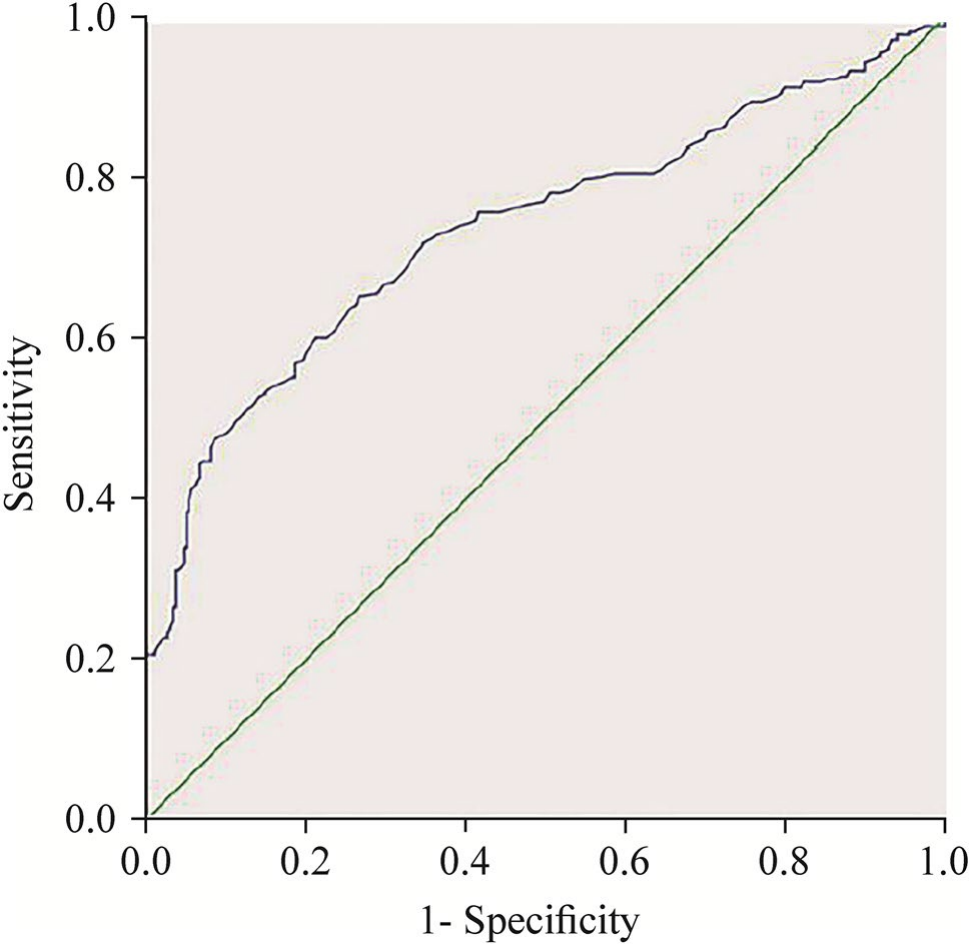
Sensitivity and specificity of BDR in the BDT. *BDR* bronchodilator response, *BDT* bronchodilator test

The ROC curve is plotted in Fig. 1. The area under the curve is 0.738. A comparison of different BDR thresholds in the bronchodilation test is available in Supplementary Table 3. The maximum value of the Youden index was 7.5%, its sensitivity and specificity were 50.7% and 87.7%, respectively. When BDR was 9%, the sensitivity and specificity were 30.9% and 94.7%, respectively (Supplementary Table 3).

## Discussion

The main pathological and physiological change of asthma patients is chronic airway inflammation. The most prominent feature of respiratory dysfunction in asthma patients is variable expiratory airflow limitation.

Airway responsiveness to bronchodilators can be used as a predictor of asthma in the childhood population [12–14]. This has been shown in various populations other than native Chinese children. In a large and populous country like China, medical resources are strained and complex and expensive tests are not widely available. Finding easy to administer and inexpensive tests is essential to get the testing and diagnostic process started at the local level. Then the treatment can be started earlier improving morbidity and reducing costs.

Bronchodilation test is a good choice, but we have been using adult indicators for a long time. The cutoff point of 12% for adult is too conservative for children. In the study carried out by Galant et al. among 51 non-asthmatic children and 346 controller-naïve asthmatic children between 4 and 17 years, the BDR value could achieve 12% in only 30.6% asthmatic children, across all severity [4]. Meanwhile, when observing BDR value in the above study, results were 2.2% [95% confidence interval (CI) 0.2–4.3] in the non-asthmatic group compared with 8.6% (95% CI 7.5–9.8) in the asthmatic group. The range seen was 7.6% (95% CI 5.8–9.5) for mild asthma to 10.1% (95% CI 7.6–12.6) in the severe persistent group. In addition, in a study [15] carried out by Harvard University among 3052 children in rural area between 8 and 15 years in Anhui Province in China, results showed BDR was 3 ± 5% in non-asthmatic children, and 7 ± 9% in asthmatic children. Moreover, in a study [6] among 142 children between 5 and 10 years in UK, 9% increase in FEV_1_ after bronchodilator use was suggested as the cutoff point with good sensitivity and specificity.

It has been shown that a persistent BDR value, even less than 12%, in asthmatic children suggests poor prognosis. In the article written by Sharma et al. [16], it showed in a 4 years study among 1041 asthmatic children in America, individuals who had a BDR of 10% had poor clinical outcomes (e.g., more hospital visits, more prednisone bursts, increased nocturnal awakenings, and missing more days of school) similar to those with a BDR of 12% and 200 mL.

The same results were also obtained in Galant et al. [17] study among 679 asthmatic children among 5–18 years.

In this study, we obtained the subjects’ information from the questionnaires which the subjects reported it by themselves or by their parents. We had a small response in FEV_1_ in the control group. We had careful screened this population but may have had some influence of general or indoor air pollution cause an increase in airway reactivity that our screening did not eliminate. Despite this the technique of using BDR to separate asthmatic from non-asthmatic children was effective.

In this study, we enrolled mild asthmatic children as these would be the ones who would likely pose the most diagnostic dilemmas. Their symptoms are atypical and not serious, often neglected by themselves and their parents, and they are easy to be missed by doctors when they see a doctor. If there is a simple and easy test to help diagnosing quickly, their asthma will be controlled more timely and their prognosis will be better. Of the 286 asthmatic children, 53.8% (154/286) had intermittent asthma, 35.3% (101/286) had mild persistent asthma, and 10.8% (31/286) had moderate persistent asthma. The results of this study showed that the BDR in non-asthmatic children was 3.30 ± 3.85%, which was consistent with the BDR results (3 ± 5%) of large-scale studies on children aged 8–15 years in rural China conducted by Kumar et al. [15]. The BDR in asthmatic children was 9.45 ± 9.15%, which was generally consistent with the results by Galant et al. [4] and Tse et al. [5].

BDR of the intermittent asthma group, mild persistent group and moderate persistent group was 9.09 ± 8.25%, 9.80 ± 10.54%, and 10.13 ± 8.84%, respectively, indicating no significant differences among the three groups. It prompts the thought that there is little difference in BDR between asthma patients with different attack frequencies and different severities.

Our results showed no relationship between BDR and gender, age, height or weight, and can be used as an independent and objective index to diagnose childhood asthma some other studies have found a relationship between BDR and gender [17–20]. The subjects of these studies were people at a special stage, these may be the influencing factors (for example, women who use contraceptives).

To evaluate the diagnostic value of a test method comprehensively and accurately, we used ROC in the analysis. The area under the ROC curve (AUC) can be understood as the average sensitivity in all specificity and reflects the diagnostic value of the method [21]. When AUC = 0.5, the method is generally considered to not work at all. When AUC > 0.7, the method is considered to have great discrimination [22, 23]. In our study, AUC was 0.738, which showed that the BDR has significant diagnostic value for asthma.

At present, the BDR threshold for asthma diagnosis in children is the same as the one for adults used widely in clinics. We found that this existing standard threshold was prone to misdiagnosis (when using BDR > 12%, the sensitivity was only 28.7% though the specificity rate of 96.3%). Many children patients with asthma were not diagnosed early, correctly and did not receive appropriate early treatment. When the BDR threshold was set to > 7.5%, the sensitivity was increased to 50.7%. At the same time, the specificity was 87.7%. This significantly improves the diagnostic accuracy of childhood asthma, reduces misdiagnosis, and prevents the children and their parents from bearing the psychological, mental and economic costs. International studies have also shown that a BDR threshold of > 12% was ineffective, and that > 9% or > 8% had better sensitivity and specificity. Although some studies have shown BDR are different in different races [24], previous results are generally consistent with ours [4–6, 25].

In summary, compared with non-asthmatic children, BDR in asthmatic children is significantly higher. BDR is not correlated with gender, age, height or weight. A BDR threshold of > 7.5% is useful in diagnosing asthma in native Chinese children.

## Electronic supplementary material

Below is the link to the electronic supplementary material.

Supplementary file1 (DOC 48 kb)
