# Supplementary material for: A clinical study to determine the threshold of bronchodilator response for diagnosing asthma in Chinese children
Source: World J Pediatr. 2019 Aug 16;15(6):559–64. doi: 10.1007/s12519-019-00293-9 (PMC6872507; doi:10.1007/s12519-019-00293-9)
Supplement: Supplementary file 1 — Supplementary file1 (DOC 48 kb) [file 12519_2019_293_MOESM1_ESM.doc]

**Supplementary Table 1**. Parameters comparison between male and female patients

| Variables | Male (*n* = 160) | Female (*n* = 141) | *P* |
| --- | --- | --- | --- |
| Age (y) | 7.52 ± 2.45 | 7.60 ± 2. 37 | 0.763 |
| Height (cm) | 129.68 ± 15.20 | 129.52 ± 16.15 | 0.930 |
| Weight (kg) | 30.49 ± 11.32 | 28.70 ± 10.60 | 0.159 |
| FEV_1_ (L) | 1.78 ± 0.58 | 1.73 ± 0.58 | 0.502 |
| FVC (L) | 1.96 ± 0.66 | 1.86 ± 0.63 | 0.195 |
| PEF (L) | 4.08 ± 1.19 | 4.15 ± 1.35 | 0.660 |
| MMEF (L) | 2.01 ± 0.72 | 2.10 ± 0.81 | 0.156 |

Values are mean ± SD. *FEV_1_* forced expiratory volume in the first second, *FVC* forced vital capacity, *PEF* peak expiratory flow, *MMEF* average flow rate of 25% to 75% out of vital capacity, *SD* standard deviation

**Supplementary Table 2**. The basic situation of different severity of asthma

| Variables | Intermittent (*n* = 154) | Mild persist (n = 101) | Moderate persist (*n* = 31) |
| --- | --- | --- | --- |
| Age (y) | 7.08 ± 2.39 | 7.38 ± 2.50 | 7.42 ± 2.51 |
| Height (cm) | 127.75 ± 15.66 | 128.98 ± 12.65 | 127.76 ± 16.29 |
| Weight (kg) | 29.75 ± 11.89 | 30.04 ± 12.65 | 30.05 ± 13.27 |
| FEV_1_ (L) | 1.49 ± 0.55 | 1.49 ± 0.54 | 1.40 ± 0.55 |
| FEV_1_ (%)-predicted | 94.89 ± 16.77 | 93.17 ± 18.31 | 90.98 ± 20.60 |
| FVC (L) | 1.78 ± 0.69 | 1.81 ± 0.69 | 1.70 ± 0.63 |
| PEF (L) | 3.47 ± 1.22 | 3.35 ± 1.17 | 2.97 ± 1.15 |
| MMEF (L) | 1.49 ± 0.59 | 1.45 ± 0.64 | 1.41 ± 0.69 |

Values are mean ± SD. *FEV_1_* forced expiratory volume in the first second, *FVC* forced vital capacity, *PEF* peak expiratory flow, *MMEF* average flow rate of 25% to 75% out of vital capacity, *SD* standard deviation

**Supplementary Table 3**. Comparison of the different thresholds of BDR on ROC curve

| Threshold of bronchodilation test | Sensitivity | Specificity |
| --- | --- | --- |
| 6% | 53.7 | 80.4 |
| 7.5% | 50.7 | 87.7 |
| 9% | 30.9 | 94.7 |
| 12% | 28.7 | 96.3 |

Values are percentage. *BDR* bronchodilator response, *ROC* receive operating characteristic curve
